# Comparative characterization of supragingival plaque microbiomes in malocclusion adult female patients undergoing orthodontic treatment with removable aligners or fixed appliances: a descriptive cross-sectional study

**DOI:** 10.3389/fcimb.2024.1350181

**Published:** 2024-05-13

**Authors:** Jiajia Zheng, Xiujing Wang, Ting Zhang, Jiuhui Jiang, Jiaqi Wu

**Affiliations:** ^1^ First Clinical Division, Peking University School and Hospital of Stomatology & National Center for Stomatology & National Clinical Research Center for Oral Diseases & National Engineering Research Center of Oral Biomaterials and Digital Medical Devices, Beijing, China; ^2^ Department of Orthodontics, Peking University School and Hospital of Stomatology & National Center for Stomatology & National Clinical Research Center for Oral Diseases & National Engineering Research Center of Oral Biomaterials and Digital Medical Devices, Beijing, China

**Keywords:** 16S rRNA gene, orthodontic brackets, clear aligners, oral plaque, oral microbiome

## Abstract

**Objectives:**

This study aimed to explore the effects of removable aligners and fixed appliances on the supragingival bacterial communities in adult female patients undergoing orthodontic treatment.

**Methods:**

Supragingival plaque samples from 48 female individuals underwent microbiome analysis (16S rRNA gene sequencing) using PacBio Sequel sequencing. The study included 13 adults without orthodontic treatment needs as the control group (Group C), and 35 patients with comparable initial orthodontic conditions who received treatment at a university clinic in Beijing, China. The treatment involved either traditional fixed brackets (Group B, n = 17) or Invisalign^®^ aligners (Group AT, n = 18). Bioinformatics methods were used for data analysis.

**Results:**

From the 48 plaque samples, a total of 334,961 valid reads were obtained, averaging 6,978 sequences per sample. The 16S rDNA sequences were classified into 25,727 amplicon sequence variants (ASVs). Significant variances in alpha and beta diversity among the groups were noted. Group B microbiome exhibited an increased presence of Gram-negative bacteria. At the phylum level, *Actinobacteriota* was significantly more prevalent in Group C samples, while *Bacteroidota* was enriched in Group B samples. Family-level relative abundance analysis showed a notable increase in *Saccharibacteria* (formerly TM7) and *Prevotellaceae* in Group B. Genus-level analysis revealed a significant rise in *Lautropia* in Group AT. Fixed orthodontic appliances were linked to oral microbiome changes, notably an enhanced relative abundance of anaerobes, including periodontal pathogens.

**Conclusion:**

The observation points to the impact of orthodontic appliance on the oral microbial community, highlighting the difference between traditional braces (Group B) and clear aligners (Group AT)in terms of the predominance of anaerobic and gram negative bacteria. This emphasizes the importance of considering the microbiological effects when choosing orthodontic appliance and underscores the need for tailored oral hygiene practices for individuals undergoing these treatments. This research might provide insights that could assist in the development of innovative cleaning techniques and antibacterial materials.

## Introduction

Developments in materials science have significantly advanced the field of invisible orthodontic technology. This technology has become increasingly popular, particularly among female patients, owing to its aesthetic appeal and comfort (Lucchese et al., 2020). One of the key advantages of these aligners is their removability, which allows for uninterrupted daily oral hygiene practices. However, the way these invisible appliances interact with enamel surface is distinct from that of traditional fixed appliances. This difference is particularly notable because patients can remove the aligners, which may impact the mechanical forces applied to the hard and soft tissues.

Clinical studies have suggested that clear aligners may be more beneficial for periodontal health than fixed appliances, potentially making them a better option for patients at risk of developing gingivitis (Jiang et al., 2018). Teenagers who use removable appliances typically demonstrate better compliance with oral hygiene and tend to accumulate less plaque compared to those wearing fixed appliances (Abbate et al., 2015). Fixed appliances, on the other hand, may facilitate plaque buildup and promote bacterial affinity to metallic surfaces (Ahn et al., 2006).

Although the current findings are informative, there is still a limited amount of research on the comparative analysis of oral microecological changes on the enamel surface environment among different orthodontic treatment method. Specifically, the effect of removable aligners on the oral microflora represents an area that requires further exploration to fully understand its implications.

With the emergence of culture-independent methods, exploring the diversity of the oral microbiota has become more feasible. Besides, the advent of third-generation sequencing (TGS) technologies, particularly those offered by the Pacific Biosciences (PacBio) platform, has simplified genome sequencing processes (Athanasopoulou et al., 2021). Combining these technologies, this study aimed to examine how removable aligners and fixed appliances affect the supragingival bacterial communities in adult female patients.

Since bacteria adhere to enamel, metal, and plastic-coated enamel surfaces in distinct ways, this research hope to offer insights that could aid in the development of innovative cleaning methods and antibacterial materials.

### Ethical approval

Ethical approval for this study was granted by the Ethics Committee of Peking University Health Science Center (PKUSSIRB-202054050). Written informed consent was obtained from all participants before their involvement in the study.

## Materials and methods

### Recruitment

The study recruited all suitable patients from October 2019 to January 2021 at the First Clinical Division, Peking University School and the Hospital of Stomatology. Participants were all female, aged between 18 and 38 years, and free from chronic periodontal disease and active caries.

Inclusion Criteria:

1. Aged above 18 years old.
2. No missing permanent teeth, except the third molars.
3. Essential oral hygiene practices. No untreated caries.
4. Good compliance, with braces worn for at least 6 months and clear aligners for at least 22 hours per day.

Exclusion Criteria:

1. Pregnancy.
2. Medical complications.
3. Lack of cooperation.
4. Chronic periodontitis and untreated dental caries.
5. Antibiotics or analgesics should be used before treatment.

Our study participants consist of 48 female patients, who are categorized into three distinct groups (**Table 1**).

**Table 1:** Demographic and clinical characteristics.

Group	Age(y)	Weight(kg)	Height(cm)
Control(N=13)	26.0 ± 6.6	58.6 ± 3.6	168.9 ± 1.9
Brackets(N=17)	26.2 ± 6.0	52.0 ± 5.9	163.4 ± 4.1
Aligners T (N=18)	27.9 ± 5.2	52.8 ± 2.3	163.3 ± 5.3

Control Group (Group C): 13 patients, serving as the control group.

Brackets Group (Group B): 17 patients underwent treatment with self-ligating fixed appliances and nickel-titanium (NiTi) archwires.

Aligners T Group (Group AT): 18 patients treated with Invisalign^®^ aligners, with samples taken from the tooth surface.

The baseline data for age, weight, and height were analyzed using one-way ANOVA tests. The results indicated no statistically significant differences among the three groups for each of these parameters: age (p=0.73), weight (p=0.79), and height (p=0.99).

### Sample collection

Supragingival plaque samples were collectedusing a sterile cotton swab. Plaque was collected from the buccal and lingual sides, as well as the occlusal surface, of the teeth ranging from 17 to 47. The orthodontic group refrained eating for 2 hours and from brushing teeth for 4 hours before plaque sampling. Samples were preserved at -80°C for subsequent analysis.

### Bacterial DNA extraction, PCR amplification, and PacBio sequel sequencing

Total DNA was extracted from the samples using the PowerSoil^®^ DNA Isolation Kit, following the manufacturer’s instructions. The 16S full-length gene was amplified using PCR with the 27F-1492R conservative region primers in a 10 μL reaction system (Solexa PCR). The specific primers used were:

Forward primer 27F: AGRGTTTGATYNTGGCTCAG
Reverse primer 1492R: TASGGHTACCTTGTTASGACTT

After constructing the sequencing library, we performed a quality check, which involved barcode identification and processing the obtained high-quality circular consensus sequencing (CCS) sequences. We then clustered the optimized CCS sequences with a 97% similarity threshold using USEARCH (version 10.0). Species classification was determined by analyzing the amplicon sequence variants (ASVs) based on their sequence composition. For species annotation and taxonomy analysis, as well as to evaluate the oral microbiota’s diversity, we employed the 16S Plaque database and the RDP Classifier. Alpha diversity analysis, examining species richness and diversity within each sample, was performed. Beta diversity analysis compared community composition and structure across samples. Metastats analysis identified significant differences at the genus level between groups, and linear discriminant analysis effect size (LEfSe) identified statistically distinct biomarkers between groups (biomarker screening criteria: LDA score>4). Various statistical techniques were utilized to establish a correlation between 16S data and the specific type of orthodontic appliance utilized by the patients.

## Results

### Taxonomic identification and relative abundance

To investigate the composition of the plaque microbial community in the three groups, plaque samples were analyzed through sequencing with the IPacBio Sequel technology A total of 334,961 valid reads were obtained from the 48 plaque samples, averaging 6,978 sequences per sample. These 16S ribosomal DNA sequences were classified into ASVs.

**Figure 1:**
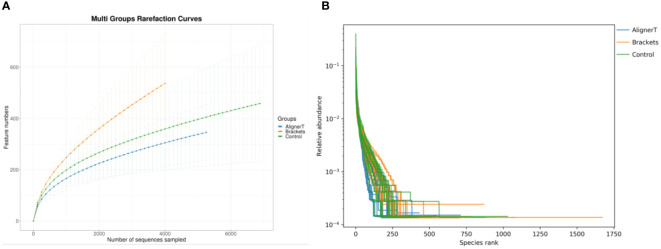
**(A)** Multi-group rarefaction curves, generated based on the number of unique amplicon sequencing variants. **(B)** Rank-Abundance Curves of Microbial Communities Under Different Conditions.

The rarefaction curves (**Figure 1**) for all groups confirmed the sufficiency of the sampling efforts.

This rank-abundance curve (**Figure 1**) underscores the variations in microbial community structure that may be attributed to the different conditions or treatments applied to each group.

### Bacterial composition

**Figure 2:**
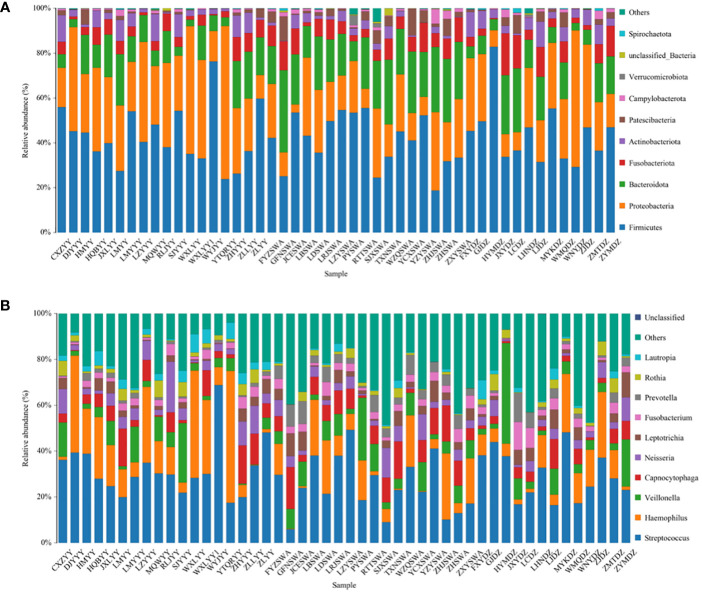
Most abundant bacterial phylum **(A)** and genus **(B)** in all samples.

The predominant phyla across all three groups were *Firmicutes* (42.9% in Group AT, 40.4% in Group B, 44.1% in Group C) (**Figure 2**). The top 10 phylum: *Firmicutes, Proteobacteria, Bacteroidota, Fusobacteriota, Actinobacteriota, Patescibacteria, Campylobacterota, Verrucomicrobiota, unclassified_Bacteria*, and *Spirochaetota*. Notably, the proportion of *Bacteroidota* increased in Group B patients. At the genus level, *Streptococcus* was the most abundant in all groups (32.0% in Group AT, 25.0% in Group B, 29.7% in Group C). Among these genera, *Streptococcus, Haemophilus, Veillonella, Capnocytophaga, Neisseria, Leptotrichia, Fusobacterium, Prevotella, Rothia*, and *Lautropia* were observed.

### Microbiome diversity and richness

#### Alpha-diversity analysis

The alpha-diversity analysis involved calculates species-level ASVs, diversity, and richness estimates for each sample. The Chao1 and ACE indices are measures of species richness, while the Shannon and Simpson index also considers species evenness.

**Figure 3:**
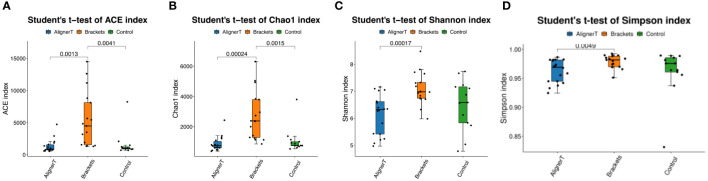
Alpha diversity was calculated based on different groups. p-values for each comparison are depicted above the box plots of the groups being compared. **(A)** ACE index. **(B)** Chao 1 index. **(C)** Shannon index. **(D)** Simpson index.

For the Chao1 index, Group C has significantly higher species richness than Group AT. The ACE index reveals significant differences in species richness between all groups, with Group AT having the lowest richness and Group C the highest. The Shannon index shows a significant difference between the AT and B groups, suggesting variance in both the richness and evenness of species between these two treatments. In Simpson index, the presence of a p-value suggests that the difference in diversity between the AT and B groups is statistically significant. The figure does not show any significant difference between the B and C groups or between the AT and C groups (**Figure 3**).

#### Beta‐diversity analysis

**Figure 4:**
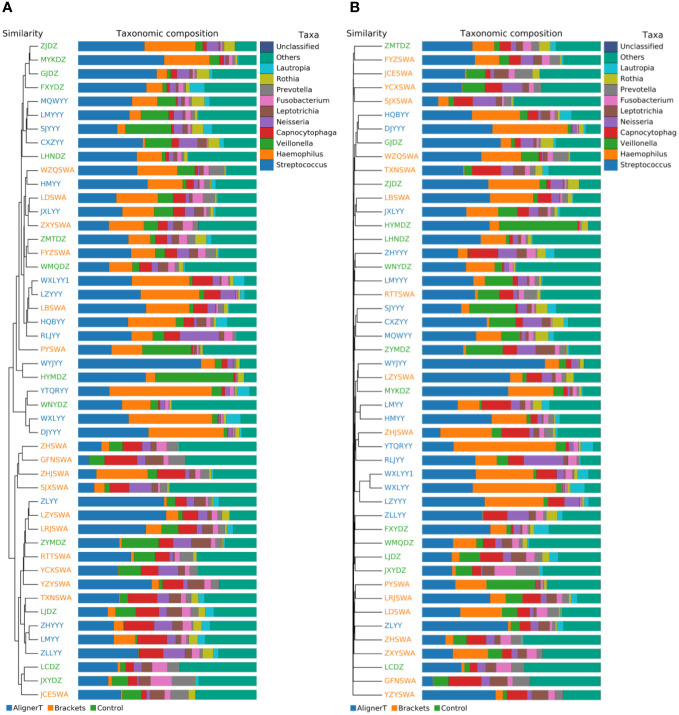
Cluster Analysis of Taxonomic Composition in Microbial Communities Across Various Sample Groups. **(A)** Weighted Unifrac. **(B)** Bray-Curtis.

Beta-diversity analysis was employed to examine and compare the community structures among the microbiotas of the three groups. Clustering analysis is presented in **Figure 4**.

The clustering in the figure displays the similarities and differences in the oral microbial communities of the patients.

**Figure 5:**
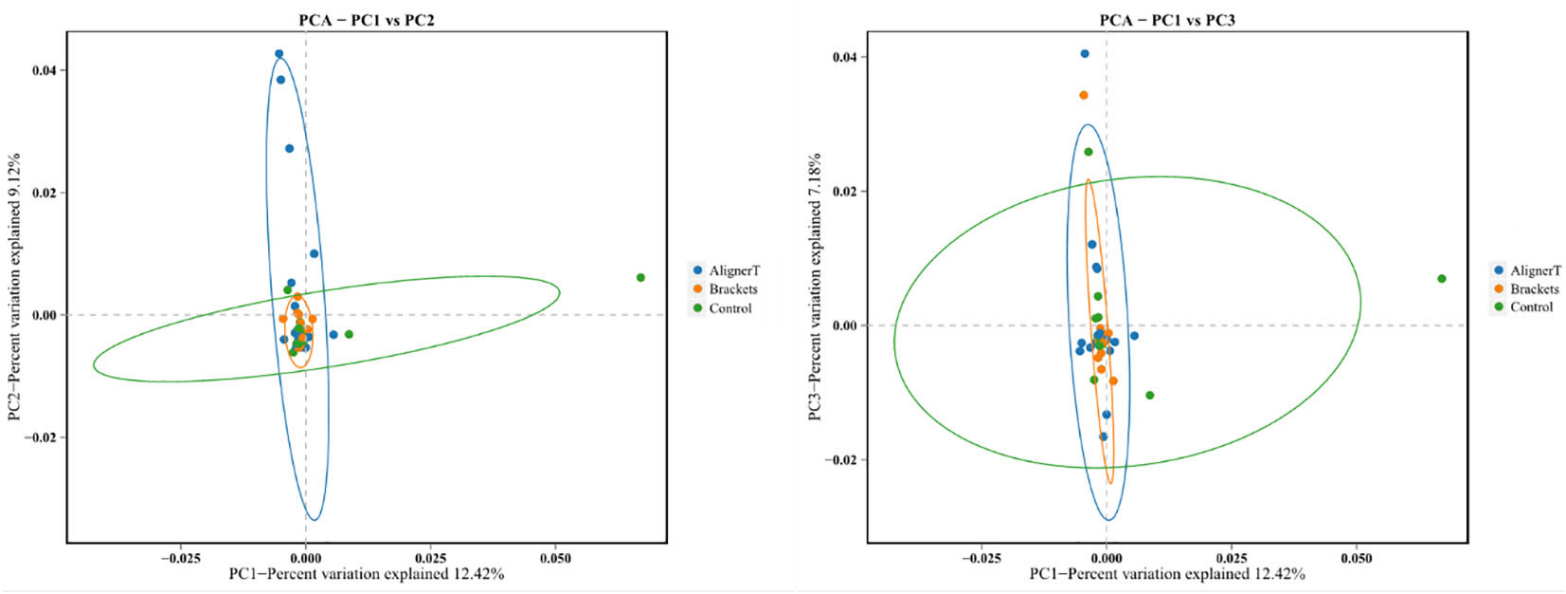
Principal components analysis using the weighted UniFrac beta-diversity metric. The ellipses denote the 95% confidence intervals, providing a visual summary of each group’s spread and indicating the variability within the groups.

**Figure 6:**
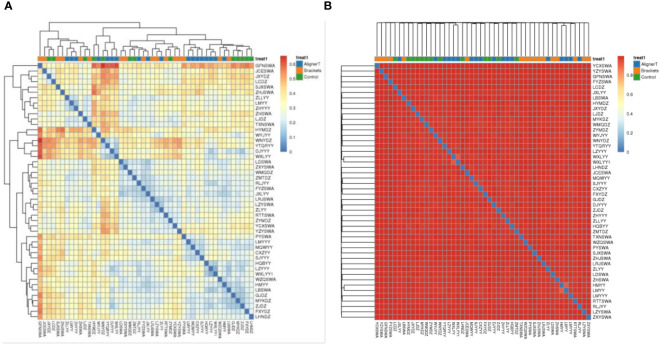
Heat map displaying correlations among 48 subjects. In the heatmap, color intensity indicates the magnitude of the correlation, with red representing positive correlations and blue indicating negative correlations. **(A)** Weighted unifrac. **(B)** Bray-Curtis.

Additionally, a principal component analysis (PCA) plot revealed no distinct separation between these groups (**Figure 5**). The relative positions of the ellipses and points indicate differences in microbial diversity or the dominance of certain taxa in one group over the other. The analysis of bacterial beta diversity showed no distinct separation between Group B and Group C. We also utilized heatmaps to visually depict the correlation coefficients among the samples (**Figure 6**).

To further investigate microbial community structure changes, we compared ASV abundance and microbial distribution at the phylum, genus, and species levels.

**Figure 7:**
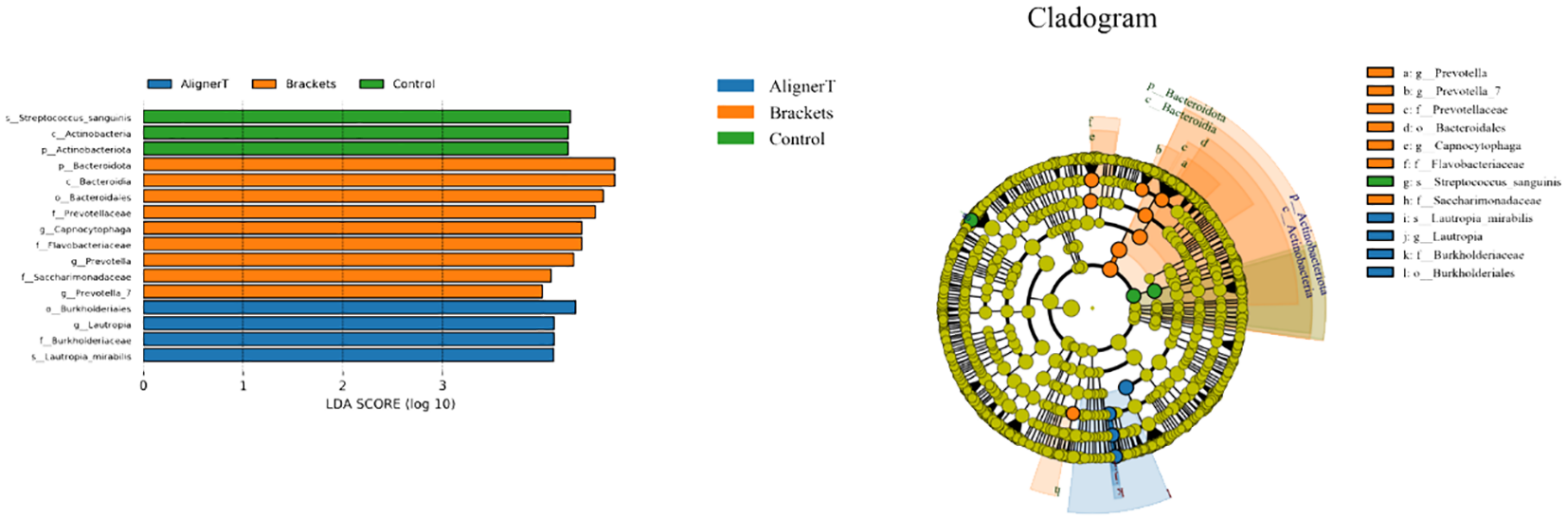
Specific taxa associated with orthodontic treatment. Linear discriminant analysis (LDA) effect size analysis of the three groups. Cladogram using LEfSe method indicating the distribution of microbes.

**Figure 7** displays the results of the LEfSe analysis, which was conducted to identify specific microbial communities. This analysis revealed 16 discriminative features (LDA>4, p<0.05) at various taxonomic levels: phylum (n=2), family (n=4), order (n=2), class (n=2), genus (n=4), and species (n=2).

At the phylum level, *Actinobacteriota* was more prevalent in Group C samples, while *Bacteriodota* was predominant in Group B.

**Figure 8:**
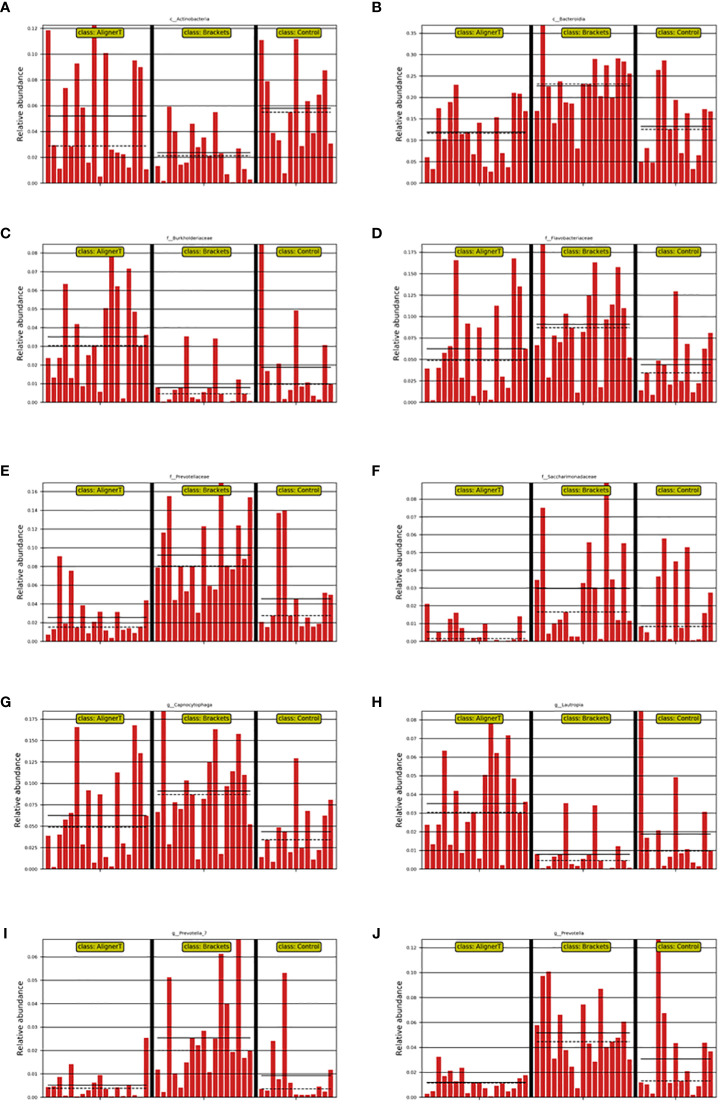
Depiction of varied microbial clade abundance in different groups: **(A)** *Actinobacteria*, **(B)** Bacteroidia, **(C)** *Burkholderiacese*, **(D)** *Flavobacteriaceae*. **(E)** *Prevotellaceae*, **(F)** *Saccharimonadaceae*, **(G)** *Capnocytophaga*, **(H)** *Lautropia*, **(I)** *Prevotella_7*, **(J)** *Prevotella.*.

**Figure 9:**
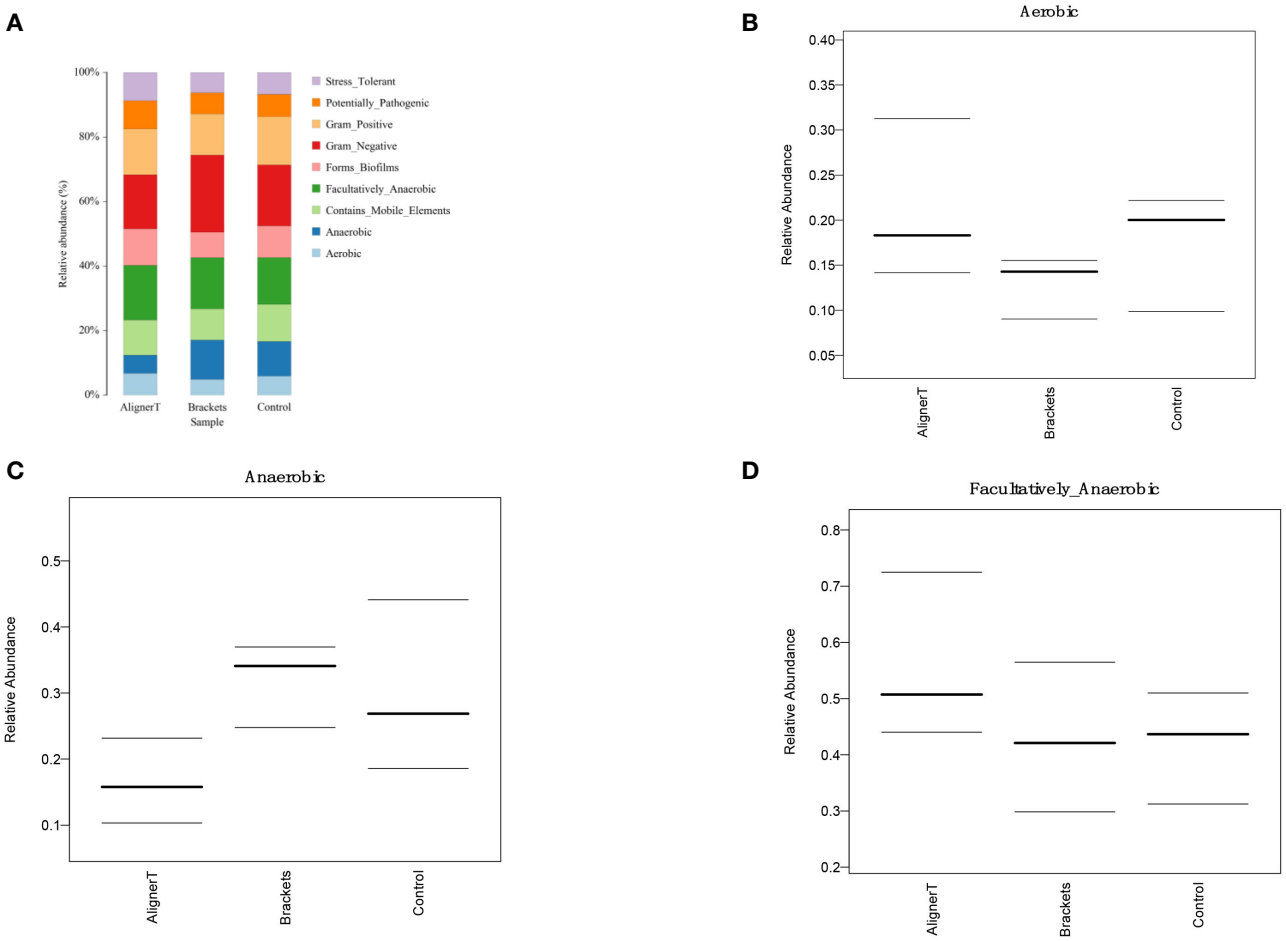
Comparative Analysis of Microbial Characteristics Across Three Groups. **(A)** the relative abundance of microbial categories. **(B–D)** plots depicting the relative abundance of aerobic, anaerobic, and facultatively anaerobic bacteria across the three groups.

Significant variations were noted among the groups in the classes *Actinobacteria* and *Bacteroidia*, families *Burkholderiaceae, Flavobacteriaceae, Saccharimonadaceae*, and *Prevotellaceae*, and genera *Capnocytophaga, Saccharimonadaceae, Lautropia, Prevotella_7*, and *Prevotella*, as depicted in **Figures 8**, **9**.

**Figure 10:**
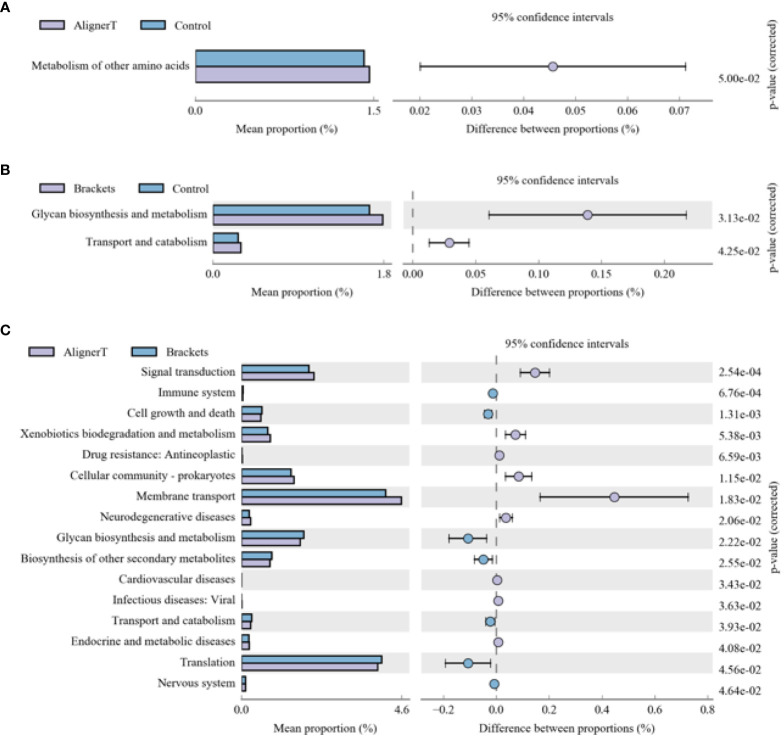
KEGG analysis results for the three groups **(A)** Group AT compared with Group C. **(B)** Group B compared with Group C. **(C)** Group AT compared with Group B.

The functional genes present in the samples were examined using the Kyoto Encyclopedia of Genes and Genomes (KEGG) database. Through differential analysis of the KEGG metabolic pathways, we can understand the differences in the functional genes of the microbial communities among different group samples in terms of metabolic pathways, as well as the extent of these variations (**Figure 10**).

The microbial communities in Group B displayed significant differences in their polysaccharide biosynthesis, metabolism, and transport when compared to those in Group C. In the case of Group AT, we observed noteworthy distinctions in the metabolism of various amino acids in comparison to Group C. From a microbial functional metabolism standpoint, the effects on the immune system, cardiovascular health, viral infections, and endocrine/metabolic disorders varied between Group AT and Group B.

**Figure 11:**
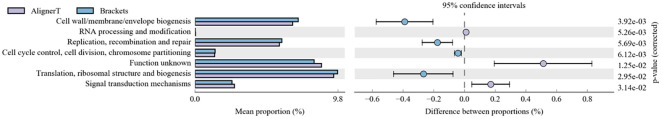
Cluster of Orthologus Groups of proteins (COG).

COGs, or Clusters of Orthologous Groups of proteins, constitute a bioinformatics resource that groups proteins from complete genomes into categories based on orthologous relationships, suggesting a common ancestral gene. We utilized this database to classify and compare the protein profiles of Group AT and Group B, as illustrated in **Figure 11**.

This figure allows for the assessment of the comparative abundance of functional protein categories between experimental conditions, highlighting areas of significant divergence that may warrant further investigation.

## Discussion

The composition of the oral microbiome is generally stable under normal conditions. However, microbiome dysbiosis, characterized by a disruption to the stability of the symbiotic microbiota, can adversely affect the host’s health status (Duran-Pinedo et al., 2021). Changes in the oral microbial environment are intimately linked to the onset and progression of periodontal diseases and caries. Existing research indicates that the use of orthodontic appliances could modify the oral cavity’s status and influence the colonization of oral biofilm by opportunistic/pathogenic strains (Perkowski et al., 2019).

The oral cavity is the entry point to the digestive tract. As such, the oral microbiome can impact the overall health and composition of the gastrointestinal (GI) microbiome (Kitamoto et al., 2020). Microorganisms present in the mouth can be swallowed and integrated into the gut microbiome. Both the oral and digestive tract microbiomes are complex ecosystems, composed of bacteria, viruses, fungi, and other microorganisms. However, the specific composition and diversity vary between these two sites (Kitamoto et al., 2020). The oral microbiome is exposed to different environmental factors (like food, oxygen levels, and oral hygiene practices) compared to the gut microbiome, which is influenced by factors like diet, medication, and intestinal conditions. Changes in the oral microbiome can have repercussions on the gut microbiome and vice versa. The oral and digestive tract microbiomes are deeply interconnected, with implications for overall health, disease, and potential therapeutic strategies.

Full-length 16S sequencing offers advantages such as longer read lengths and more expansive amplification regions, enhancing the comprehensiveness of the analysis (Hong et al., 2024). This approach allows for a more precise species classification and a broader scope of species annotation compared to traditional 16S sequencing methods used in second-generation platforms. In this study, third-generation sequencing technology was utilized to achieve full-length sequencing. To reduce potential biases due to variations in sex hormone levels, the study exclusively included adult female subjects.

It has been shown that the salivary and teeth surface microbiota of patients with invisible braces undergo changes 12 hours post-application of clear aligners (Yan et al., 2021). Saliva and dental plaques are primary sample sources reflecting the oral microbial community. Dental plaque provides a more accurate representation of the bacteria that adhere to tooth surfaces and contribute to dental caries and periodontitis (Campobasso et al., 2021). Studying bacteria within this biofilm environment is crucial for understanding their behavior, and interactions, which differs significantly from their planktonic (free-floating) counterparts in saliva (Jakubovics, 2015). Owing to the difficulty in accessing dental plaques, only a limited number of studies have employed them as sources of samples. Our study used supragingival dental plaque and found an average of 6978 ASVs per patient. We characterized the microbiomes of Groups B and AT patients and compared them to those of orally healthy controls (Group C).

*Firmicutes, Planctomycetota, Bacteroidota*, and *Cyanobacteria* emerged as dominant bacteria across the three groups. *Firmicutes* are involved in the initial colonization of the tooth surface and the formation of dental plaque. Some species, like *Streptococcus mutans* produce acids from carbohydrate fermentation, contributing to tooth decay (Homayouni Rad et al., 2023). As a key etiological agent of human dental caries, *Streptococcus mutans* primarily resides in dental plaque (Wang et al., 2019). They have a remarkable ability to metabolize fermentable carbohydrates, especially sucrose, to produce lactic acid, which lowers the pH in the mouth and leads to the demineralization of the tooth enamel (Abranches et al., 2018).

For alpha diversity index, **Figure 3** collectively suggest that there are differences in microbial diversity associated with the different orthodontic treatments, as indicated by several biodiversity indices. The Group AT consistently shows lower diversity compared to the Group C, and there are noticeable differences between the Group AT and B groups. Clear aligners, made from thermoplastic material, are removable and fit over the teeth. The thermoplastic material itself may influence the oral environment differently than traditional braces, affecting bacterial growth.

The PCA plots function as a means to visualize complex microbial data and simplify the comparison of microbial communities across various treatment groups. In **Figure 5**, the ellipses that overlap indicate a similarity between the microbial communities in Group AT and Group B. Moreover, the beta diversity—which indicates variation in microbial community structure—reveals no significant differences between Group C and Group B. This may be related to the fact that invisible braces fully envelop the tooth surface. In contrast, the tooth surfaces in the fixed orthodontic group are exposed to the oral environment.

We found the presence of orthodontic brackets can create unique niches and environmental conditions in the oral cavity. The environments where dental plaque forms are different; there is plaque accumulation beneath bracket slot, brackets, and under archwires. According to our research findings, fixed orthodontics may create a more anaerobic environment on the enamel surface.

Fixed orthodontic appliances pose challenges to oral hygiene and may temporarily impact periodontal health (Azaripour et al., 2015). Research indicates increased microbial diversity in patients with fixed orthodontics compared to normal individuals (Sun et al., 2018).These conditions may favor certain bacterial species or alter bacterial behavior. A meta-analysis suggests that patients treated with Invisalign^®^ exhibit better periodontal health than those with traditional fixed appliances (Lu et al., 2018). Previous studies have highlighted that Invisalign^®^ aligner margins are typically designed below the marginal gingiva, and their smooth surface, coupled with a maximum usage duration of 14 days per aligner, results in significantly less biofilm accumulation compared to traditional fixed orthodontic and removable appliances (Zhao et al., 2020).

In Group B, there was a significant reduction in *Actinobacteriota* compared to Groups AT and C. *Actinobacteriota*, formerly known as *Actinobacteria*, is a phylum of Gram-positive bacteria. While certain bacteria within the *Actinobacteriota* phylum may be present in the oral cavity, their direct association with dental caries is not as prominent as that of other bacteria like mutans streptococci (from the *Firmicutes* phylum) (Qudeimat et al., 2021). Brackets can make oral hygiene more challenging, leading to changes in the microbial community. The reduction in *Actinobacteriota* might reflect an imbalance in the oral microbiome due to these changes.

At the family level, higher levels of *Flavobacteriaceae, Prevotellaceae*, and *Sacchariomonadaceae* were observed in Group B. *Flavobacteriaceae* specific role in oral health is less clear. *Prevotellaceae* is commonly found in the human oral cavity and gut. They are anaerobic and are known to play a role in the breakdown of proteins and carbohydrates. Increased levels of *Prevotellaceae* in the oral microbiome are often associated with periodontal disease (Tian et al., 2021). The alteration in the microbiome composition, particularly the increase in families associated with periodontal pathogens, underscores the importance of enhanced oral hygiene practices for patients with fixed braces. In Group C, because the absence of orthodontic appliances, the natural balance of the oral microbiome, including *Actinobacteriota*, is expected to be maintained.

Within Group AT, elevated levels of the family *Burkholderiaceae* were observed. *Burkholderiaceae* is not commonly associated with the oral cavity (Kerber-Diaz et al., 2022). However, advancements in microbial sequencing have started to reveal a more diverse array of bacteria in dental plaque than previously recognized. Their presence in dental plaque might also indicate a broader diversity in the oral microbiome than traditionally understood. Insights into lesser-known bacterial families in the oral cavity could lead to the development of targeted therapeutic strategies for managing oral health.

Longitudinal studies have identified biofilm maturation and caries lesion progression with an increase in Gram-negative anaerobes, including *Veillonella* and *Prevotell*a (da Costa Rosa et al., 2021). Our observation that higher levels of *Prevotella* and *Prevotella_7* were observed at the genus level in Group B suggests a significant shift in the oral microbial community among those wearing fixed orthodontic appliances. These changes in the oral environment can create conditions that favor the growth of *Prevotella* species, as they thrive in anaerobic conditions and can utilize the nutrients available in the accumulated dental plaque. An increase in *Prevotella* levels might indicate a higher risk of periodontal disease or caries development (Sharma et al., 2022), making it important for individuals with fixed braces to maintain rigorous oral hygiene and have regular dental check-ups. *Streptococcus, Lactobacillales*, and *Neisseria* cinerea were also noted. This aligns with another study that found Neisseria mucosa had higher levels at sites with orthodontic bands either at (OBM) or below the gingival margin (OBSM) (Kim et al., 2010). The significant increase in *Lautropia* in Group AT suggests that the use of the clear aligners creates an oral environment that favors the growth or proliferation of *Lautropia.* While *Lautropia* is not typically associated with oral diseases (Keijser et al., 2018), its increased prevalence could indicate changes in the oral ecosystem that might have implications for oral health, although these would likely be benign. *Saccharimonadaceae* falls within the realm of bacterial taxonomy. It’s part of a more extensive classification that includes various bacteria with differing characteristics and roles. While some families like *Streptococcaceae* and *Actinobacteriota* are well-known for their roles in oral health and disease, the role of *Saccharimonadaceae* in dental plaque might be less clear (Tian et al., 2022). The next-generation sequencing allowed us to uncover of this previously underexplored microbial resident in the supragingival plaque. Its role in oral health still needs to be further explored.

Changes in polysaccharide biosynthesis, metabolism, and transport suggest a shift in how bacteria process and utilize carbohydrates. This could be due to altered food retention around the brackets, changes in saliva flow, or variations in the oral microbiome composition.

Microecological alterations in the oral environment can lead to detrimental changes in the composition or metabolic activity of the oral microbiome (Nascimento et al., 2019). The specific mention of differences in amino acid metabolism indicates that the bacteria present in Group AT might be processing amino acids differently compared to the normal oral microbiome. This could be due to changes in pH, oxygen levels, or nutrient availability caused by the aligners.

The oral microbiome can modulate the immune response (Hou et al., 2022). A balanced microbiome supports immune function, while dysbiosis (microbial imbalance) can trigger inflammatory responses, potentially leading to systemic effects. Research has suggested connections between oral health and cardiovascular diseases (Carasol et al., 2023). For example, periodontal disease has been associated with an increased risk of heart disease (Priyamvara et al., 2020). The oral microbiome can influence the body’s resistance to or susceptibility to viral infections. A healthy microbiome can act as a barrier to infection, while an imbalanced one might increase vulnerability (Di Stefano et al., 2022). It highlights the need for a holistic approach in orthodontics, considering not just dental outcomes but also broader health implications.

These findings highlight the need to understand how orthodontic treatments influence the oral microbial ecosystem. This knowledge can guide dental professionals in recommending appropriate oral hygiene practices for patients with braces or aligners. It also contributes to the development of materials and designs that minimize adverse effects on the oral microbiome.

A notable limitation of this study is its sole focus on adult female patients. This specific demographic inclusion restricts the applicability of the results to a more diverse patient population, potentially affecting the generalizability of the findings.

## Limitation

One notable limitation of our study is the exclusion of male subjects, potentially impacting the generalizability of our findings.

## Conclusion

The observation points to the impact of orthodontic appliance on the oral microbial community, highlighting the difference between traditional braces (Group B) and clear aligners (Group AT) in terms of the predominance of anaerobic and gram negative bacteria. This emphasizes the importance of considering the microbiological effects when choosing orthodontic appliance and underscores the need for tailored oral hygiene practices for individuals undergoing these treatments. This research might provide insights that could assist in the development of innovative cleaning techniques and antibacterial materials.

## Data availability statement

The datasets presented in this study can be found in online repositories. The names of the repository/repositories and accession number(s) can be found below: NCBI, under BioProject PRJNA1064555 (http://www.ncbi.nlm.nih.gov/bioproject/1064555).

## Ethics statement

The studies involving humans were approved by Ethics Committee of Peking University Health Science Center (PKUSSIRB-202054050). The studies were conducted in accordance with the local legislation and institutional requirements. The participants provided their written informed consent to participate in this study.

## Author contributions

JJ: Investigation, Writing – review & editing, Supervision, Resources. XW: Writing – review & editing, Supervision, Validation. TZ: Writing – review & editing, Methodology, Supervision, Software. JZ: Writing – original draft, Software, Formal analysis, Data curation. JW: Writing – review & editing, Writing – original draft, Supervision, Resources, Project administration, Funding acquisition, Conceptualization.
